# The millipede family Cryptodesmidae in Indochina (Diplopoda, Polydesmida)

**DOI:** 10.3897/zookeys.578.7994

**Published:** 2016-04-07

**Authors:** Sergei I. Golovatch

**Affiliations:** 1 Institute for Problems of Ecology and Evolution, Russian Academy of Sciences, Moscow, Russia

**Keywords:** Diplopoda, Cryptodesmidae, taxonomy, new genus, new species, Vietnam, Laos

## Abstract

In addition to two species of the diplopod family Cryptodesmidae already reported from Indochina, *Trichopeltis kometis* (Attems, 1938) and *Ophrydesmus anichkini* Golovatch, 2015, the fauna of that region is supplemented by three species: *Niponia nodulosa* Verhoeff, 1931, a millipede hitherto known only from southern Japan and Taiwan, is now recorded from Vietnam; *Trichopeltis cavernicola* **sp. n.** from Laos, the sixth species in that tropical Asian genus, is the first presumed troglobite to be described amongst the Asian cryptodesmids and shows several distinct troglomorphic features; and *Circulocryptus* **gen. n.**, monobasic, which joins the tribe Dyakryptini, but differs from all three contribal genera (two monobasic from Borneo, and another, oligotypic, from New Guinea) primarily in the gonopods of *Circulocryptus faillei* **sp. n.**, from Vietnam, being especially elaborate and subcircular, the telopodites strongly twisted, and the solenomere lying much more basally.

## Introduction

In tropical or subtropical Asia and Australasia, the millipede family Cryptodesmidae currently comprises only 11 genera (including two that are dubious) and 34 species. Among them, only two genera and species, *Trichopeltis kometis* (Attems, 1938) (= *Trichopeltis deharvengi* Golovatch, Geoffroy, Mauriès & VandenSpiegel, 2010) and *Ophrydesmus anichkini* Golovatch, 2015, occur in Indochina, the former species being quite widespread in Vietnam, Laos and Cambodia, the latter species described from a single locality in southern Vietnam (Golovatch 2015, Golovatch and Akkari 2016).

This paper puts on record another three Cryptodesmidae found in Indochina. One represents a new genus and species from Vietnam. One more is a new species of a rather small tropical Asian genus from a cave in Laos, the first presumed troglobitic Asian cryptodesmid. The third species is common in southern Japan and Taiwan, being reported here for the first time from Indochina, in particular, northern Vietnam.

## Material and methods

The types of both new species were collected by my French colleagues and friends Louis Deharveng and Anne Bedos, both from the Muséum national d’Histoire naturelle, Paris (MNHN), France, and Arnaud Faille, from the Bayerische Zoologische Staatssammlung, München (ZSM), Germany, and are now deposited in MNHN. The samples of *Niponia nodulosa* Verhoeff, 1931, were taken by friend Dmitri N. Fedorenko (Moscow, Russia), and are now housed in the Zoological Museum of the Moscow State University, Moscow (ZMUM), Russia. Focus stacking pictures were taken by Jonathan Brecko, of the Royal Museum for Central Africa, Tervuren (MRAC), Belgium.

## Taxonomic part

### Niponia nodulosa

Taxon classificationAnimaliaPolydesmidaCryptodesmidae

Verhoeff, 1931

#### Material.

1 ♂, 1 ♀ (ZMUM ρ 3115), Vietnam, 25 km E of Hai Phong, Cat Ba Island, National Park, ca 10 km road-km NW of Cat Ba City, N20°47‘56“, E106°59‘47“, 10-24.X.2011, leg. D. Fedorenko.

#### Remarks.

Based on material from Taiwan, this species has recently been redescribed and illustrated in due detail (Golovatch et al. 2011). It is common in southern Japan and Taiwan (Golovatch et al. 2011), erroneously reported also from continental China (Golovatch et al. 2010). The Vietnamese samples fully agree with the detailed account given by Golovatch et al. (2011).

### Trichopeltis cavernicola sp. n.

Taxon classificationAnimaliaPolydesmidaCryptodesmidae

http://zoobank.org/61A071B5-A30A-49D4-B10D-A6833E8343D9

Figs 1–3 , 4–7

#### Type material.

Holotype ♂ (MNHN JC 367), Laos, Khammouane Prov., Ban Naden, Cave Tham Namlat, N17.504969°, E105.385598°, ca 180 m a.s.l., 09.11.2011, leg. L. Deharveng & A. Bedos (Lao 11-51).

#### Name.

To emphasize the cavernicoly and troglomorphic appearance of this new species; noun in apposition.

#### Diagnosis.

Differs from congeners by the relatively gracile and slender body in which each midbody paratergite is clearly narrower than prozonite width, coupled with the increasingly upturned postcollum paraterga and such obviously troglomorphic features as an unpigmented body, as well as the extremely long and slender legs and antennae. In addition, much of the distolateral part of an indistinctly tripartite gonopod telopodite is densely covered with wart-shaped knobs while the tip of the solenomere is vestigial and simple.

#### Description.

**Figures 1–3.**
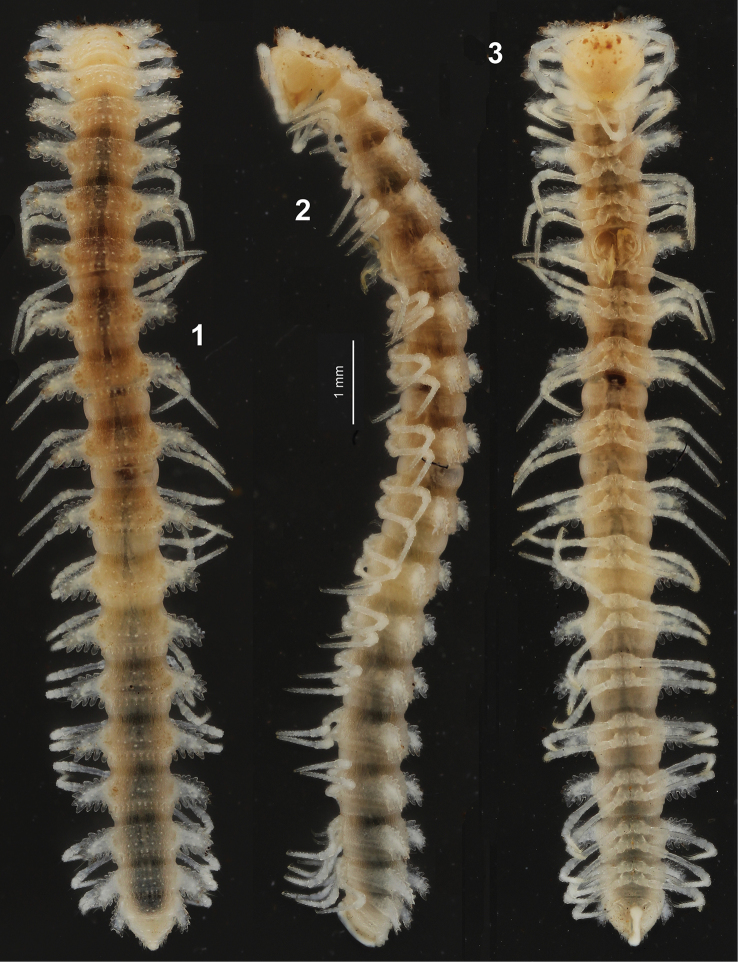
Habitus of *Trichopeltis cavernicola* sp. n., ♂ holotype, dorsal, lateral and ventral views, respectively.

Length ca 11 mm, width of midbody pro- and metazonae 0.8 and 1.7 mm, respectively. General coloration in alcohol entirely pallid, tegument mostly translucent (Figs 1–3).

Body with 20 segments (composed of collum plus 17 podous and one apodous rings, plus telson). In width, head << collum < segment 2 < 3 < 4–17; thereafter body rapidly tapering towards telson. Head behind labrum very densely and clearly microgranulate and pilose; epicranial suture superficial, more distinct in vertigial region. Antennae unusually long and slender for a cryptodesmid (Figs 2, 3), barely clavate, not placed inside a groove, in situ reaching behind segment 3 when stretched laterally or ventrolaterally; in length, antennomere 2 > 5=6 > 3=4; antennomeres 5 and 6 each with a compact apicodorsal group of bacilliform sensilla. Interantennal isthmus about as broad as diameter of antennal socket. Tegument dull, clearly microgranulate almost throughout, only prozonae usually more finely shagreened. Dorsum from only slightly and regularly convex to increasingly flat towards midbody metazonae; paraterga mostly set at about upper 1/4 of body, very strongly developed (Figs 1–3), beginning with collum. Collum completely covering the head from above, biconvex, but clearly lobulate anteriorly (10+10 lobulations) and caudolaterally (5+5 lobulations, including lateralmost one shared with anterior row), but not mediocaudally; paraterga distinctly upturned as opposed to subhorizontal paraterga 2-7. Postcollum paraterga short and narrowly rounded laterally, on each side usually with 3-4 lateral, 4-5 anterior and 4-5 posterior lobulations, gradually, but increasingly upturned and elevated above dorsum towards segment 19 (Figs 1–3). Marginal lobulations on paraterga mostly rounded, relatively large and well separated from one another, lateral end of paraterga drawn caudad gradually, but increasingly on segments 15–19, produced behind rear tergal margin only on segments 18 and 19. Caudal row of lobulations on paraterga gradually diminished in size towards paratergal base to get transformed into 5+5 clearly smaller, subequal, subhorizontal, caudomarginal lobules, each of the latter bearing a smaller, narrowly rounded, subvertical tubercle/knob at base. Another two, more or less regular, transverse rows of 6-8+6-8 similar, almost sharp, subvertical knobs extending onto paraterga in front of mediocaudal row until about segment 15, followed by three irregular rows of similar knobs thereafter (Fig. 1). As a result, middle parts of metaterga 2–14 with three, of following metaterga with four, transverse, usually rather irregular rows of small, round, setigerous knobs/tubercles in addition to caudalmost, regular row of smaller lobules just in front of a microspiculate limbus. Tergal setae long and simple, mostly abraded, only a few retained on several caudal metaterga (Fig. 1). Neither axial line nor radii, nor pleurosternal carinae. Ozopores invisible, pore formula untraceable. Stricture dividing pro- and metazonae broad, shallow and microgranulate like adjacent metazonae. Epiproct (Figs 1–3) short, conical, subtruncate at apex. Hypoproct (Fig. 3) subtrapeziform, 1+1 caudal setae clearly separated, borne on small knobs.

**Figures 4–7.**
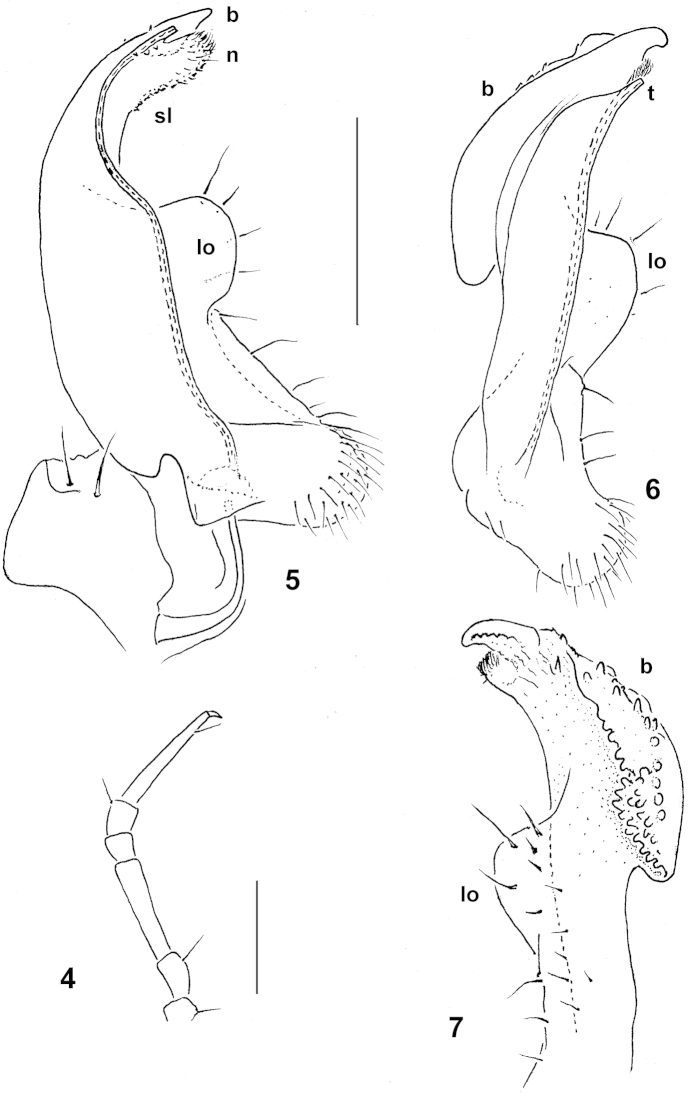
*Trichopeltis cavernicola* sp. n., ♂ holotype: **4** leg 7, lateral view **5–7** left gonopod, mesal, submesal and sublateral views, respectively. Designations: **lo** lobe at midway of telopodite; **sl** solenomere branch; **t** tip of solenomere; **b** lateral branch; **n** setose lobule Scale bars: 0.2 mm.

Sternites narrow (Fig. 3), sparsely setose, moderately impressed along main axis, clearly broadened only between coxae 7 and 9, respectively. Legs very long and slender, without modifications (Figs 1–4), ca 1.4–1.5 times as long as paratergal width (♂) (Figs 1–3); in length, femora = tarsi >> prefemora > coxae > postfemora and tibiae (Fig. 4); gonapophyses on coxae 2 small cones; neither adenostyles nor tarsal brushes. Gonopod aperture transversely ovoid, caudal and lateral margins thin, slightly elevated (Fig. 3).

Gonopods (Fig. 5–7) fairly complex, in situ held parallel to each other (Fig. 3). Telopodite only slightly curved caudad, vaguely tripartite. A short, simple, very sparsely setose, rounded lobe (**lo**) lying caudally at about midlength of telopodite and marking the end of the latter’s stout half. A twice as slender acropodite subdivided into an irregularly shaped lateral branch (**b**), longest, subunciform apically and densely beset with wart-like knobs on lateral face, and a rather simple, suberect, mesal solenomere branch (**sl**) apically extended into a small, mesal, densely setose lobule (**n**) with an adjacent, minute, simple, rod-shaped solenomere proper (**t**). Neither an accessory seminal chamber nor a hairy pulvillus.

#### Remarks.

*Trichopeltis* Pocock, 1894 (= *Otodesmus* Cook, 1896), formerly comprised six species that range from the Himalayas of India (Assam and Darjeeling District) and Myanmar to southern China, Indochina and Indonesia (Sumatra) (Golovatch et al. 2010, Golovatch and Akkari 2016).

The new species is not only the first clearly troglomorphic *Trichopeltis*, but also the first presumed troglobite among the Asian Cryptodesmidae. Similarly strongly cave-adapted cryptodesmids are exceptional, e.g. the highly troglomorphic *Peridontodesmella alba* Schubart, 1957, from several caves in São Paulo State, Brazil (Trajano et al. 2000).

### Circulocryptus gen. n.

Taxon classificationAnimaliaPolydesmidaCryptodesmidae

http://zoobank.org/B7688BED-6280-4700-A7F3-F3CA32A02952

#### Diagnosis.

Cryptodesmidae with gonopod telopodite stout, subcircular, clearly twisted (= seminal groove mostly running on lateral face) and vaguely bipartite, supplied with a mesal prefemoral process at base; a considerable, spiniform, retrorse solenomere terminating a sigmoid seminal groove and placed near telopodite midway; neither an accessory seminal chamber nor a hairy pulvillus.

#### Name.

To emphasize the subcircular gonopods; gender masculine.

#### Description.

Small-sized Cryptodesmidae (adults about 1 cm long and 3 mm wide) with 20 segments (trunk composed of collum plus 17 podous and one apodous rings, plus telson); a flabellate collum covering the head from above. Antennae clearly clavate. Paraterga short and very broad, slightly declined, mostly squarish laterally, lobulate and/or radiate anteriorly, laterally and posteriorly; ozopores invisible, pore formula untraceable. Middle parts of metaterga with seriate transverse rows of abundant setigerous knobs/tubercles partly extending onto paraterga; tergal setae present, simple; neither sternal cones nor leg modifications. Gonopod aperture subcordiform; gonopod telopodites in situ held parallel to each other, each vaguely bipartite and complex, with a mesal, parabasal, prefemoral process; basal part of telopodite clearly twisted and surmounted by a long, spiniform solenomere terminating a sigmoid seminal groove and situated at about telopodite midway. Neither an accessory seminal chamber nor a hairy pulvillus.

#### Type-species.

*Circulocryptus faillei* sp. n.

#### Remarks.

Globally, the Cryptodesmidae is a relatively small family that encompasses almost 40 genera and nearly 130 species and ranges from Mexico to Argentina in the Americas, occurring also in tropical Africa and tropical to subtropical Asia to Papua New Guinea and Japan in the East (Minelli 2015). Only three genera have hitherto been known in the entire family, in which the gonopod shows a distinct prefemoral process arising mesally near the origin of the seminal groove: *Astrolabius* Verhoeff, 1931, with 2 species from Papua New Guinea (Golovatch et al. 2010), *Dyakryptus* Hoffman, 1961, monobasic, from Eastern Malaysia, Sabah, Borneo (Hoffman 1961, 1980), and *Sarissocryptus* Hoffman, 1993, monobasic, from Eastern Malaysia, Sarawak, Borneo (Hoffman 1993). This process can be completely mesal (*Astrolabius*) or be twisted laterad (*Dyakryptus* and *Sarissocryptus*). Based of the above synapomorphy in gonopod structure, i.e. the presence of a basal prefemoral process, coupled with a coherent distribution pattern in Borneo and New Guinea, these three genera form the tribe Dyakryptini Hoffman, 1973 (Golovatch 2015). *Circulocryptus* gen. n. definitely joins this trio, but differs in the gonopods being especially elaborate and subcircular (versus far from so elaborate and not too strongly curved), the telopodites distinctly twisted basally (versus not twisted), and the solenomere lying much more basally, close to telopodite midlength (versus subapical). The range of Dyakryptini is thus considerably extended into continental SE Asia.

### Circulocryptus faillei sp. n.

Taxon classificationAnimaliaPolydesmidaCryptodesmidae

http://zoobank.org/BAAD23EF-944F-4698-BBE5-9FD3FF9E66A2

Figs 8–9 , 10–12

#### Type material.

Holotype ♂ (MNHN JC 368), Vietnam, Khanh Hoa Prov., Cat Lam Distr., Suoi Cat community, Hon Ba Nature Reserve, road to Alexandre Yersin‘s monument in Hon Ba, 1350-1550 m a.s.l., mountain tropical rainforest, 14.XI.2013, leg. A. Faille (Vn No. 298).

#### Name.

Honours Arnaud Faille, the collector.

#### Description.

**Figures 8, 9.**
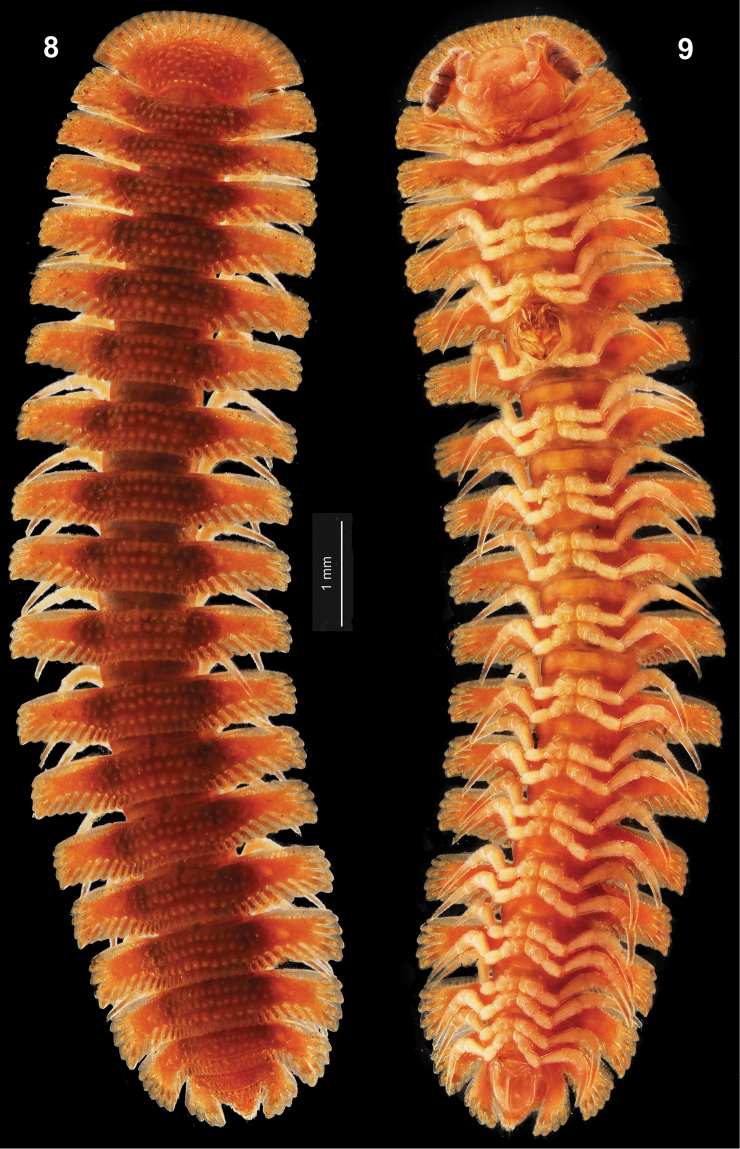
Habitus of *Circulocryptus faillei* sp. n., ♂ holotype, dorsal and ventral views, respectively.

Length ca 11 mm, width of midbody pro- and metazonae 1.2 and 2.9 mm, respectively. General coloration in alcohol red, but legs mostly somewhat lighter pink and antennomeres 5-7 increasingly infuscate, red-brown to dark brown (Figs 8, 9).

Body with 20 segments. In width, head << collum < segment 2 < 3 < 4=15; thereafter body rapidly tapering towards telson.

Head behind labrum very densely and clearly microgranulate; epicranial suture superficial, more distinct in vertigial region. Antennae short, clearly clavate (Fig. 9), C-shaped and placed mostly inside a groove; in length, antennomere 3 = 5 = 6 > 4 > 1 = 2 = 7; antennomeres 5 and 6 each with a compact apicodorsal group of bacilliform sensilla. Interantennal isthmus almost twice as broad as diameter of antennal socket. Tegument dull, clearly microgranulate almost throughout, only prozonae more finely shagreened. Dorsum moderately and regularly convex; paraterga mostly set at about upper 1/4 of body, very strongly developed (Figs 8, 9), beginning with collum, usually only slightly declined ventrad. Collum flabellate, completely covering the head from above, clearly radiate only anteriorly (12+12 long, apically setigerous radii), fore margin regularly convex, non-lobulated, but a distinctly and regularly emarginated caudal margin with 5+5 small setigerous lobulations; dorsal surface irregularly beset with small, dense, roundish, setigerous tubercles/knobs (Fig. 8). Postcollum paraterga very broad, each clearly broader than prozona; lateral edge mostly subquadrate, drawn increasingly behind rear tergal margin only in segments 16–19; paraterga 19 produced as far behind as tip of epiproct. Fore margin/shoulder of paraterga straight, with numerous short radii, but lateral and caudal margins slightly, but sufficiently distinctly lobulated, lateral edge with an abbreviated anterolateral and 4 or 5 fully developed lateral lobulations; caudal edge increasingly poorly, but abundantly radiate and lobulate toward base. Mid-dorsal parts of metaterga typically with 3 transverse, more or less regular rows of small, rounded, low tubercles/knobs in front of 5-7+5-7 smaller lobulations forming a regular row at caudal margin between main axis and bases of paraterga; 1–2 rows of dorsal knobs growing increasingly obliterated when extended onto central parabasal parts of paraterga. Each marginal lobulation/radius and tergal tubercle normally surmounted by a short, poorly visible seta, these setae often being obliterated. Neither axial line nor pleurosternal carinae. Ozopores invisible, pore formula untraceable. Stricture dividing pro- and metazonae broad, shallow and microgranulate like adjacent metazonae, but shining. Epiproct (Figs 8, 9) short, conical, subtruncate at apex. Hypoproct (Fig. 9) subtrapeziform, 1+1 caudal setae clearly separated, borne on small knobs.

**Figures 10–12.**
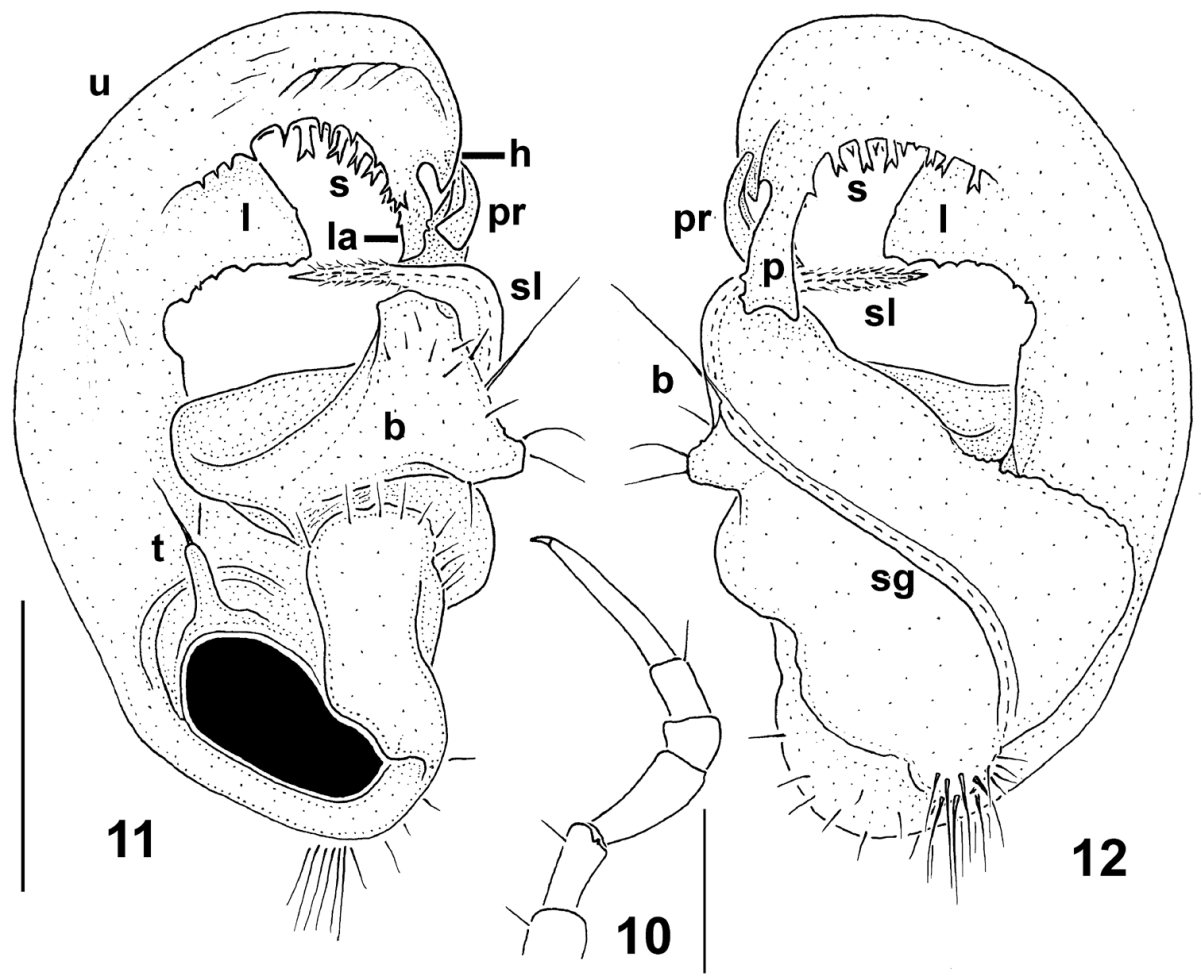
*Circulocryptus faillei* sp. n., ♂ holotype: **10** leg 7, lateral view **11, 12** left gonopod, mesal and lateral views, respectively. – Scale bars: 0.25 mm (10) and 0.2 mm (11, 12). Designations: **sg** seminal groove; **t** prefemoral process; **b** basal part of telopodite; **sl** solenomere; **pr** apical process of **b**; **u** distal half of telopodite; **l** midway lobe; **s** simple or bifid spinules; **p** lateral spatuliform process; **la** mesal subunciform lappet; **h** ventral hook at base of **la**. NB: the large black spot in Fig. 11 is a condyle.

Sternites narrow (Fig. 9), sparsely setose, moderately impressed along main axis, somewhat or very clearly broadened only between coxae 7 and 9, respectively. Legs unmodified, long, but relatively stout (Figs 8–10), ca 1.4–1.5 times as long as paratergal width (♂); in length, tarsi > femora > prefemora and tibiae > coxae and postfemora (Fig. 10); gonapophyses on coxae 2 small cones (Fig. 9); neither adenostyles nor tarsal brushes. Gonopod aperture subcordiform, caudal and lateral margins slightly elevated (Fig. 9).

Gonopods (Figs 11, 12) very complex, in situ both held parallel to each other (Fig. 9). Telopodite subcircular, very strongly curved caudad, vaguely bipartite, at very base with a short, prefemoral, finger-shaped, trichostele-like process (**t**). Basal part (**b**) stout, extended terminally into a long, solid, spiniform, retrorse, laterally curved and densely pilose solenomere (**sl**), the latter at its base bearing a short, slightly curved, apical process (**pr**); seminal groove (**sg**) sigmoid, mostly running on lateral face of **b**, this being evidence of **b** torsion. Acropodite about half as long as telopodite, supplied with a subtriangular, midway, membranous lobe (**l**) and a large apical uncus (**u**) showing two caudal, parallel and strongly adjacent rows of simple or bifid spinules (**s**) before **u** branching into two peculiar apical structures, one a lateral spatuliform process (**p**) (in situ directed strongly laterad), the other a mesal subunciform lappet (**la**) with a smaller ventral hook (**h**) at base. Neither an accessory seminal chamber nor a hairy pulvillus.

## Supplementary Material

XML Treatment for Niponia nodulosa

XML Treatment for Trichopeltis cavernicola

XML Treatment for Circulocryptus

XML Treatment for Circulocryptus faillei
